# Mitigation of sulfide adsorption in natural gas by silanized stainless steel: insights from density functional theory

**DOI:** 10.1038/s41598-025-04484-5

**Published:** 2025-07-29

**Authors:** Huadong Zhu, Li Zhou, Pu Zhang, Ahmad Umar, Sotirios Baskoutas, Wen Zeng

**Affiliations:** 1 Natural Gas Research Institute of PetroChina Southwest Oil & Gas Field Company, Chengdu, 610213 China; 2 Key Laboratory of National Market Supervision (Natural Gas Quality Control and Energy Measurement), Chengdu, 610213 China; 3 Key Laboratory of Natural Gas Quality Control and Energy Measurement, China National Petroleum Corporation, Chengdu, 610213 China; 4 Department of Chemistry, Faculty of Science and Arts, and Promising Centre for Sensors and Electronic Devices (PCSED), Najran University, 11001 Najran, Kingdom of Saudi Arabia; 5 STEM Pioneers Training Lab, Najran University, 11001 Najran, Kingdom of Saudi Arabia; 6 Department of Materials Science and Engineering, The Ohio State University, Columbus, OH 43210 USA; 7 Department of Materials Science, University of Patras, 26500 Patras, Greece; 8 College of Materials Science and Engineering, Chongqing University, Chongqing, 400030 China

**Keywords:** Sulfur compounds, Silanization stainless steel, Adsorption Inhibition, Density functional theory, Engineering, Materials science, Physics

## Abstract

This study employs Density Functional Theory (DFT) to investigate the inhibition of sulfide adsorption in natural gas through the use of silanized stainless steel. Various adsorption models were developed to explore the interactions between hydrogen sulfide, thionyl carbon, methyl mercaptan, and ethyl mercaptan with silanized stainless steel surfaces. Calculations revealed positive adsorption energies of 1.55 eV, 1.87 eV, 1.56 eV, and 1.60 eV, respectively, indicating a non-spontaneous adsorption process. Furthermore, the adsorption configurations of these sulfur compounds on the surface closely resembled their free states, indicating weak interactions with the surface. Charge population analysis indicated minimal charge transfer between the sulfides and the silanized stainless steel, suggesting dissociative chemical adsorption is unlikely. Experimental results, corroborated by theoretical predictions, demonstrate that the silanization coating serves as an inert shield for stainless steel, effectively resisting sulfide adsorption. A 60-day adsorption test confirmed stable concentrations of sulfide gases with minor fluctuations, validating the efficacy of silanization treatment in hindering sulfide adsorption.

## Introduction

Sulfur compounds like hydrogen sulfide, methyl mercaptan, ethyl mercaptan, and thionyl carbon pose safety risks^1,2^, induce pipeline corrosion, and impacting on natural gas quality. Consequently, monitoring these compounds throughout the stages of gas production, distribution, storage, and sales becomes paramount. Total sulfur and hydrogen sulfide content, particularly in gas quality standards, serve as pivotal metrics^3–7^. Yet, due to the propensity of sulfur compounds to adsorb to pipelines, valves, and sampling equipment, their accurate measurement proves challenging. To mitigate this issue in sulfur compound detection within natural gas, ISO 10715 recommends using glass and polytetrafluoroethylene (PTFE) as sampling containers^8^. However, glass is fragile and unsafe for high-pressure sampling, while PTFE is permeable to certain gases like helium and hydrogen^8^. Silianization surface treatment technology, as a novel method, possesses lower adsorption characteristics and chemical inertness. This technique employs silanes to create a protective film on metal surfaces, bonding chemically to provide a stable and consistent layer. This unique protective layer exhibits robust resistance against sulfide adsorption^9–13^, consequently, silanized stainless steel has gained widespread use in gas sampling and sulfide analysis. Nevertheless, despite its evident practical effectiveness, and some corrosion inhibitor to stainless steel was reported^14,15^, there remains a dearth of research elucidating the specific mechanisms and principles governing its resistance to sulfur adsorption.

Theoretical methods employed in adsorption studies encompass empirical, semi-empirical approaches, and first-principles ab initio calculations. While empirical and semi-empirical methods suit larger system simulations, they may lack depth in explaining physical processes^16–18^. On the flip side, first-principles ab initio calculations, rooted in the Schrödinger equation, offer a more profound understanding of substance properties. Despite their computational complexity and limitations with larger systems, their significance in theoretical research has grown, aided by computational advancements and various approximation techniques. Notably, first-principles Density Functional Theory (DFT) has become the predominant approach in this domain^10,19^. DFT, leveraging electron density, adeptly simulates and forecasts adsorption phenomena, prized for its thoroughness, high precision, and innovation. Researchers have carried out a lot of research work on gas molecules such as H_2_S, COS, CH_3_SH and so on. There have been some reports on the theoretical aspect, especially from the microscopic point of view of the electronic structure. Hao et al. investigated the adsorption mechanism of H_2_S and CH_3_SH on Fe (110) surface^20^. The results show that This also shows that in oil and gas, H_2_S, CH_3_SH and S atoms generated after dissociation are very easy to adsorb on the surface of iron pipes, which directly affects the accuracy of the determination of sulfide gas in oil and natural gas. Ren et al. analyzed the adsorption properties and bond strength of H_2_S on Fe(1 0 0) surface by means of density functional theory. The results showed that a new chemical bond was formed between H_2_S molecule and matrix^21^. Fabiani et al. studied the adsorption of H_2_S, HS, S and H on the Fe (310) step surface, and compared the adsorption results with that on the Fe (100) surface. The results showed that the adsorption of HS, S and H on the bridge, quadruple hollow and triple hollow sites were relatively stable^22^. Saraireh et al. calculated the adsorption and dissociation paths of CH_3_SH molecules at highly symmetrical adsorption sites on Fe surface using exact DFT with van der Waals correction. The stability order of dissociated products is (CH_4_, S) > (CH_3_, S, H) > (- SCH_3_, H) > (–CH_3_, SH)^23^.However, it grapples with computational complexity, accuracy issues under specific conditions, and limitations in application scope^24–29^. Presently, researchers have harnessed DFT to explore adsorption structures and electronic behaviors of sulfur compound gas molecules on pure α-Fe_2_O_3_(001) and vacant α-Fe_2_O_3_(001) surfaces^30^. They have also optimized the geometric structure of H_2_S through DFT methods, calculating the quantity of H_2_S molecules adsorbed onto stainless steel surfaces^31^. Nonetheless, there remains a gap in DFT-focused research delving into the adsorption mechanism of sulfides in natural gas on silanized stainless steel surfaces.

This study pioneers the use of DFT computational methods to explore how silanized stainless steel hinders sulfide adsorption in natural gas. Employing Materials Studio software, it delves into calculating and scrutinizing the adsorption energy of the most stable configurations, evaluating alterations in surface state energy due to adsorption, and employing charge difference density to delve deeper into interface interactions. Furthermore, the research delves into bonding and dissociation post-sulfide adsorption while corroborating findings through experimental validation from both macroscopic and microscopic viewpoints. This comprehensive approach provides vital theoretical backing for the development of sulfur-resistant adsorption materials.

## Theoretical calculation

### Microscopic characterization of silanized steel bottle slices

In utilizing Materials Studio for adsorption energy calculations, the initial phase involves acquiring the microscopic layout of the silanized steel surface. This entails gathering details about surface features like dislocations, defects, and orientations, vital for constructing a precise adsorption surface model. Following this, the Cambridge Sequential Total Energy Package (CASTEP) module within Materials Studio is utilized to compute the adsorption energy.

**Fig. 1:**
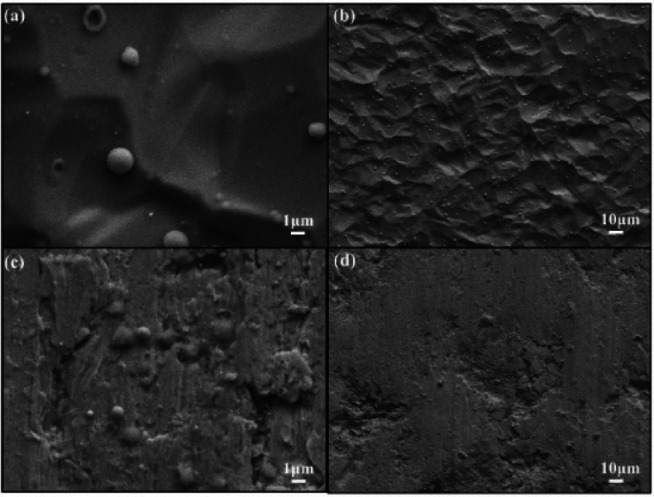
SEM morphology of (**a**) and (**b**) silanized stainless steel surface and (**c**) and (**d**) stainless steel bottle surface.

Gathering data about surface features like dislocations, defects, and orientations on silanized steel involves cutting silanized stainless steel bottles using wire cutting to create 1 cm × 1 cm block samples. These samples serve for surface characterization and to facilitate adsorption experiments under varied conditions. Examining scanning electron microscope (SEM) images (Fig. 1) unveils uniformly dispersed spherical objects across the surface.

**Fig. 2:**
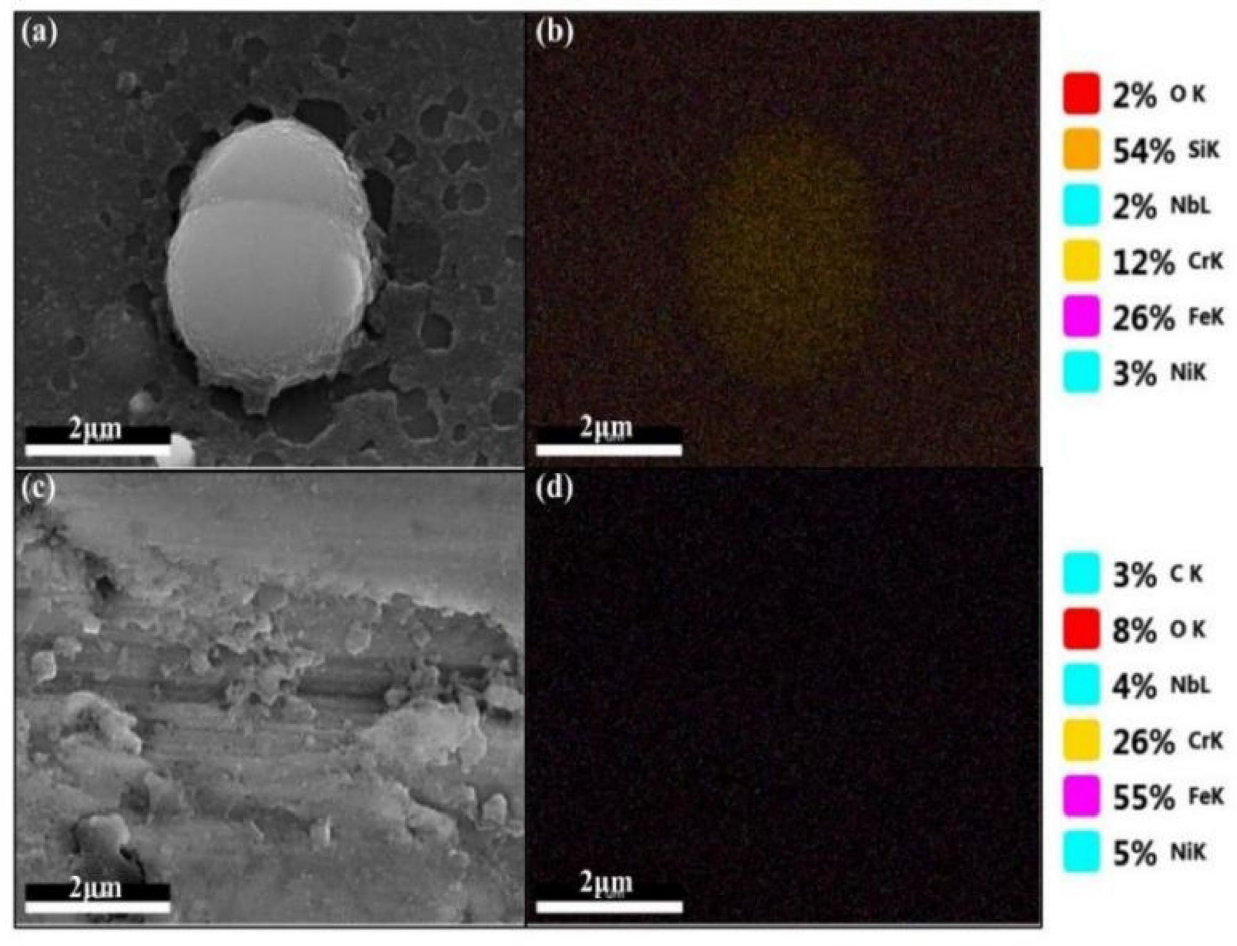
SEM morphology and EDS energy spectra of (**a**) and (**b**) local area of silanized stainless steel surface and (**c**) and (**d**) local area of stainless steel bottle surface.

The Energy-Dispersive X-ray Spectroscopy (EDS) surface scan results (Fig. 2) clearly indicate that silicon is the primary component constituting the spherical objects adhered to the surface of the silanized stainless steel samples. Hence, it can be deduced that these spherical residues originate from the silanization process.

**Fig. 3:**
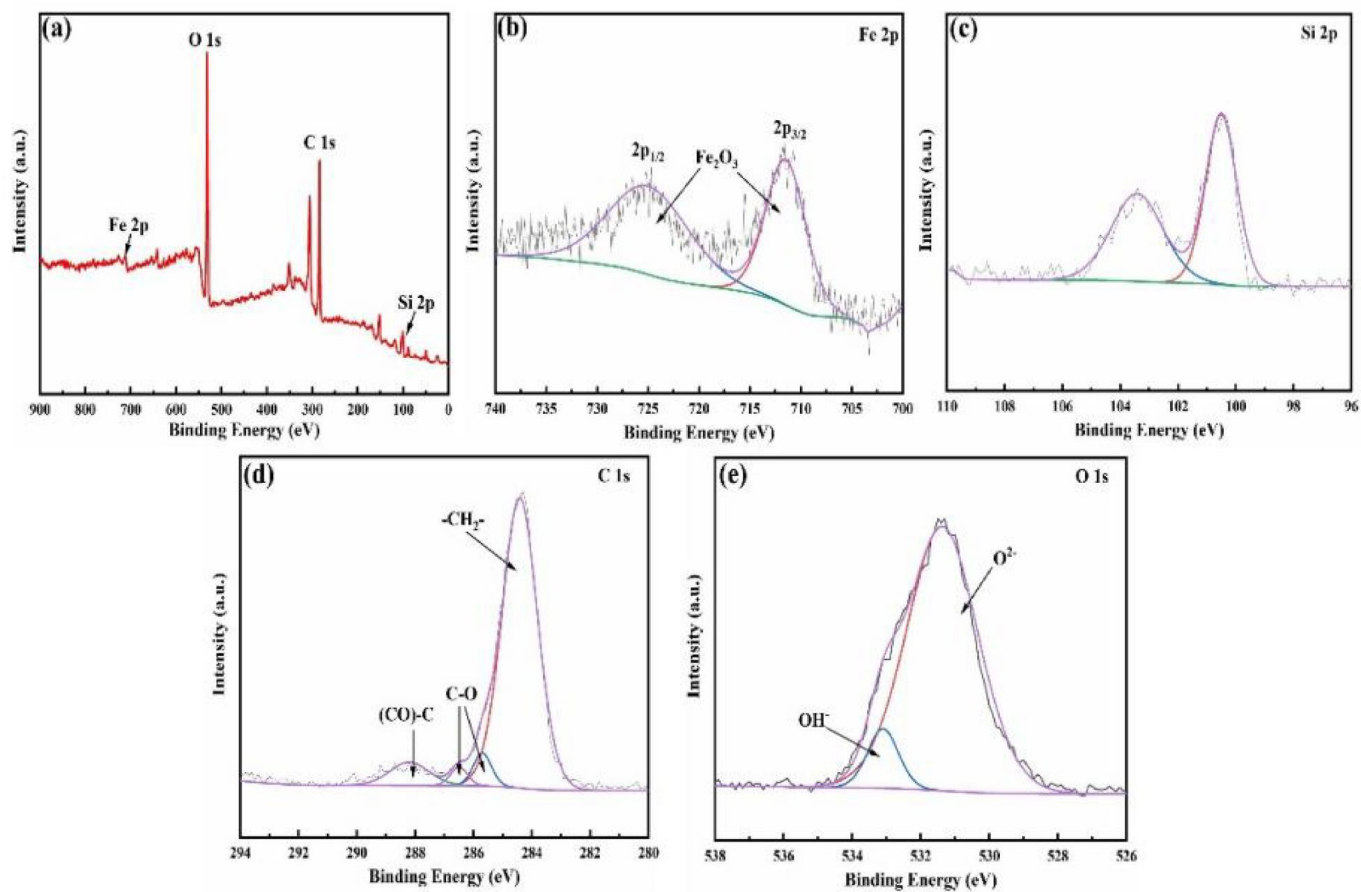
XPS spectra of silanized stainless steel surface: (**a**) survey, (**b**) Fe 2p, (**c**) Si 2p, (**d**) C 1s, and (**e**) O 1s.

For a more precise establishment of the silanized stainless steel surface model for subsequent investigations, X-ray Photoelectron Spectroscopy (XPS) was employed to further analyze the elemental composition and chemical states. In Fig. 3a, the comprehensive survey spectrum of the silanized stainless steel surface affirms the presence of Si, Fe, O and C elements. Figure 3b zooms in on the high-resolution view of Fe 2p, showcasing the characteristic peak of Fe oxide at a binding energy of 710.9 eV. Figure 3c reveals a peak corresponding to silicon oxide at a binding energy of 103.5 eV, indicating the potential presence of silica on the surface. Additionally, Fig. 3d illustrates two distinct peaks at 286.5 eV and 285.5 eV associated with C-O bonds, while peaks at 284.5 eV and 288.1 eV correspond respectively to the CH_2_- group and (CO)-C, integral components of surface silane molecules. In the O 1s spectrum, the presence of metal oxides and OH- groups is suggested by the appearance of two characteristic peaks at 531.2 eV and 533.1 eV.

### Construction of the silanized stainless steel adsorption model

Initially, the inherent crystal cell structure of Fe underwent optimization via Materials Studio software, producing a stable model. Subsequently, a static calculation was conducted on this model, providing information regarding the energy bands and electronic state density of intrinsic Fe. The optimization process yielded an energy value of -55565.765 eV for the stainless steel.

Referring to the austenitic stainless steel structure, particularly the γ-Fe (111) surface, and considering elemental analysis along with XPS findings, it was established that silane establishes Si-O-Fe covalent bonds on the surface. Additionally, silane molecules, via the condensation reaction of Si-OH groups, generate a distinct silane film featuring Si-O-Si groups on the stainless steel surface. Utilizing these characteristic groups as a foundation, a model representing the surface of silanized stainless steel was developed.

The visualizer module within Materials Studio was utilized to create gas molecule models for four sulfides: hydrogen sulfide, carbonyl sulfide, methanethiol, and ethanethiol. To undergo geometric optimization, these models were situated within a three-dimensional cubic unit cell measuring 10 Å per side. As these are discrete small molecules, a 1 × 1 × 1 Brillouin zone k-point sampling method was chosen.

## Adsorption configuration, adsorption energy, and charge population analysis

**Fig. 4:**
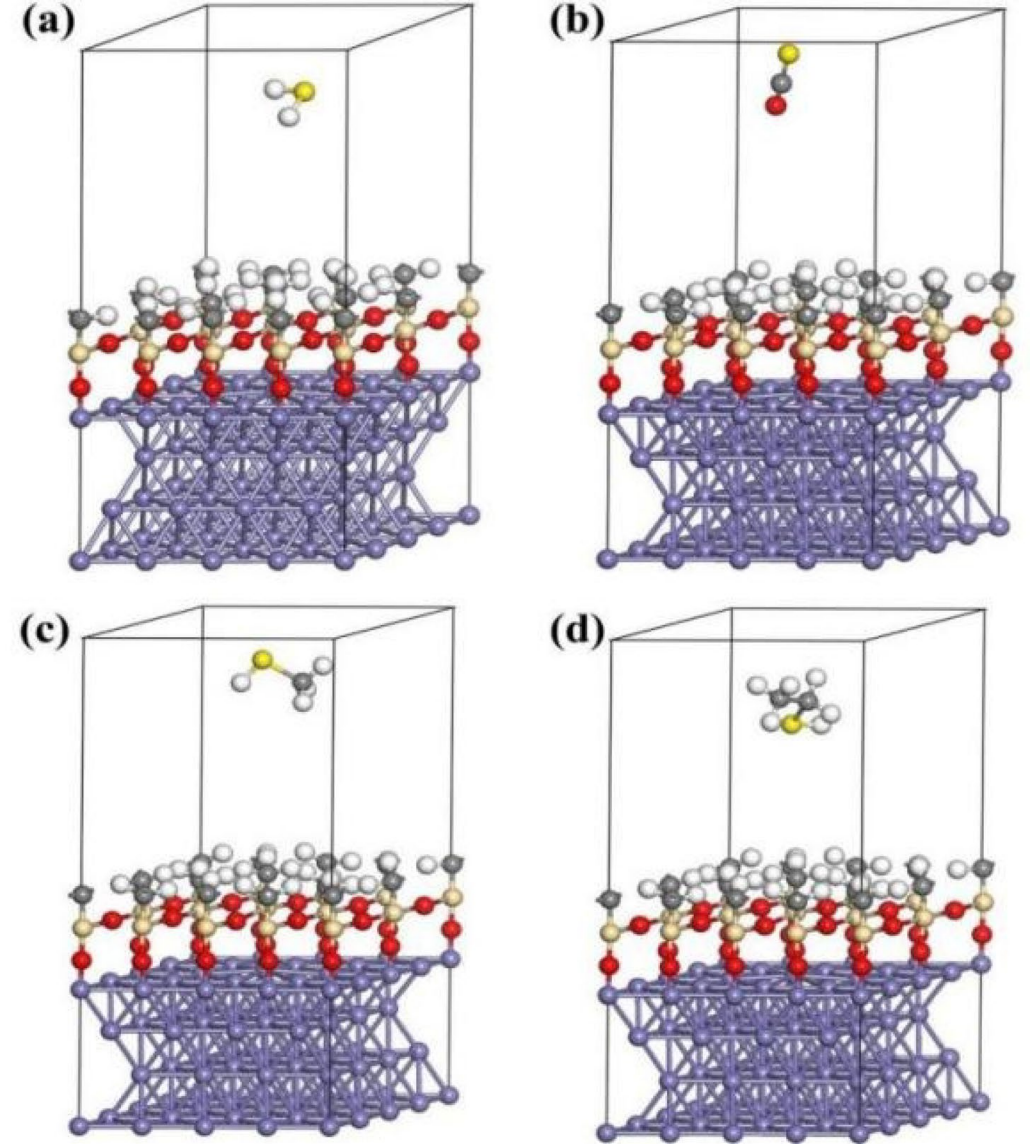
Optimized molecular model diagram on silanized stainless steel surface: (**a**) H_2_S, (**b**) COS, (**c**) CH_3_SH, and (**d**) C_2_H_5_SH.

The silanized stainless steel surface, coated with a layer of silanized atomic structure, does not possess distinct symmetry points. Hence, in establishing the adsorption model, simply situate the gas molecules on its surface suffices. This computational procedure mirrors that employed with stainless steel. The four sulfide molecule variants were positioned 3 Å perpendicular to the model of the silanized stainless steel surface. Throughout the structural optimization, all four types of sulfide molecules showcased a tendency to shift away from the silanized stainless steel surface (Fig. 4).

**Table 1:** Adsorption energy of sulfide molecules on silanized stainless steel surface.

Adsorbed molecules	H_2_S	COS	CH_3_SH	C_2_H_5_SH
Adsorption energy (eV)	1.55	1.87	1.56	1.60

As per Table 1, the positive values for the adsorption energies of all sulfide molecules signify that the adsorption process elevates the system’s energy, necessitating external energy input. Consequently, the adsorption is not spontaneous. As depicted in Table 2, the configurations of the adsorbed gas molecules (bond lengths and angles) remain nearly unaltered in comparison to their free state, indicating a weak interaction between the gas molecules and the surface.

**Table 2:** Structural characteristic parameters of the adsorption of four sulfide molecules on silanized stainless steel surface.

Adsorption system	Bond length (Å)		Bond angle (°)	
H_2_S	S-H_1_	1.372	H_1_-S-H_2_	90.596
COS	C-O	1.177	S-C-O	179.98
	C-S	1.624		
CH_3_SH	S-H_1_	1.357	H_1_-S-C	94.984
	S-C	1.853	S-C-H_2_	108.60
	C-H_2_	1.097	S-C-H_3_	107.97
	C-H_3_	1.100	S-C-H_4_	111.27
	C-H_4_	1.097		
C_2_H_5_SH	S-H_1_	1.354	H_1_-S-C_1_	97.015
	S-C_1_	1.837	S-C_1_-C_2_	109.282
	C_1_-C_2_	1.522	S-C_1_-H_2_	108.55
	C_1_-H_2_	1.100	S-C_1_-H_3_	108.64
	C_1_-H_3_	1.101	C_1_-C_2_-H	103.56
	C_2_-H_4_	1.102		
	C_2_-H_5_	1.101		
	C_2_-H_6_	1.101		

As indicated in Table 3, the total charge change values for the four sulfides hover close to 0, indicating minimal charge transfer between them and the adsorbent substrate. This suggests a repulsive interaction between the silanized surface and the four sulfide molecules, diminishing the likelihood of chemical adsorption. By amalgamating insights from adsorption configuration, adsorption energy, and charge population analysis, it is foreseeable that these four sulfide molecules will not adhere to the silanized stainless steel surface.

**Table 3:** Mulliken charge population analysis of sulfide molecule adsorption on silanized stainless steel surface.

Adsorbed molecules	H_2_S	COS	CH_3_SH	C_2_H_5_SH
Q(e)	0.02	0	0.02	0.01

## Sulfur-resistant adsorption verification experiment

### Microscopic adsorption verification experiment

1 cm × 1 cm samples of silanized stainless steel were prepared and positioned within a 500 mL adsorption container. Over a continuous 20-day period, a standard substance mixture containing 20 mg/m^3^ of methanethiol, carbonyl sulfide, hydrogen sulfide, and ethanethiol was consistently introduced. Following this duration, the samples underwent microscopic characterization to confirm the adsorption mechanism foreseen through theoretical calculations.

**Fig. 5:**
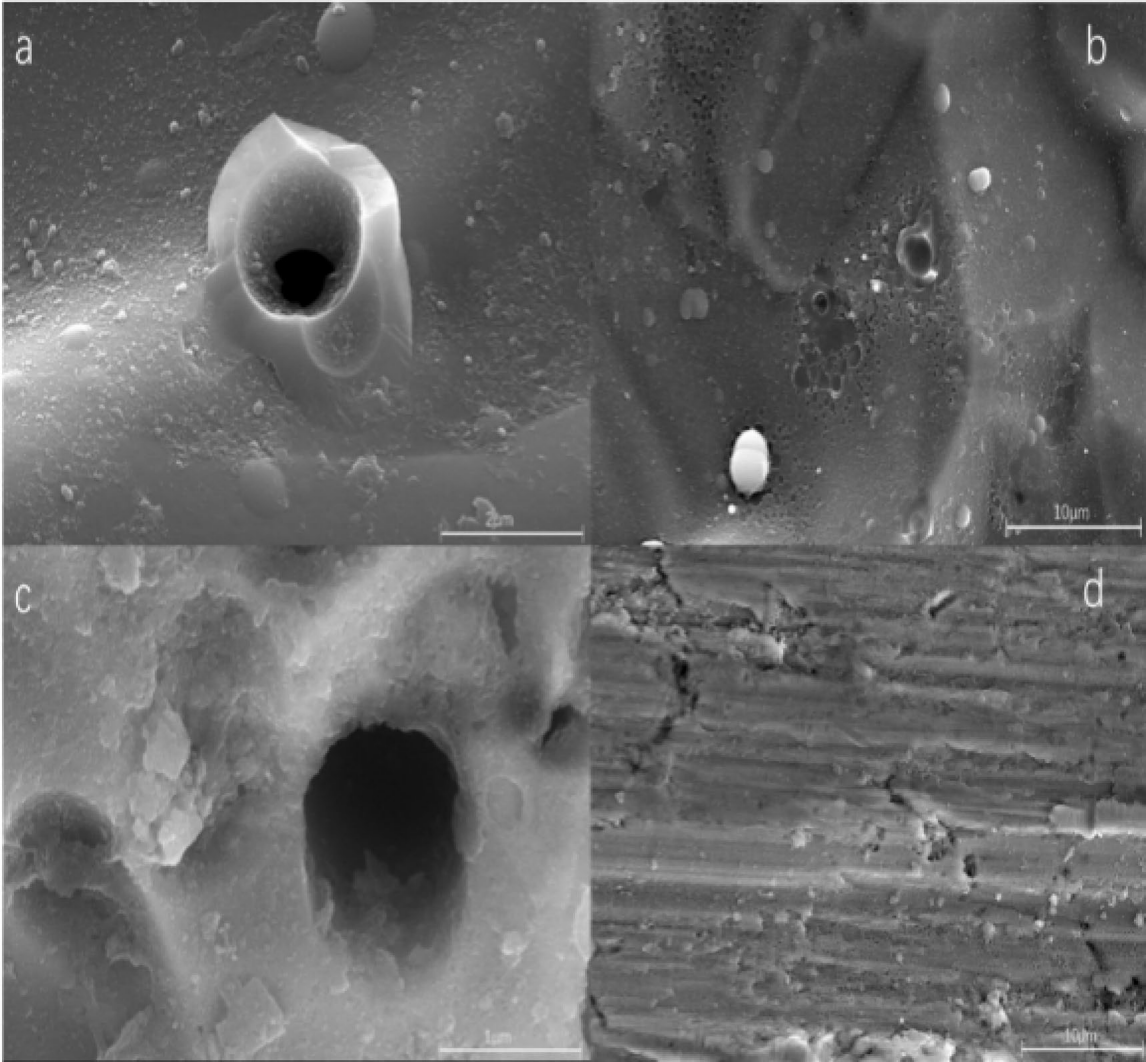
SEM morphology of (**a**) and (**b**) silanized stainless steel surface and (**c**) and (**d**) stainless steel bottle surface.

For microscopic characterization to assess surface structural alterations pre- and post- adsorption, SEM was employed (Fig. 5). Results revealed minimal surface changes before and after adsorption. The silanized stainless steel surface maintained its relative smoothness, showcasing the formation of a dense film that offered protective coverage.

**Fig. 6:**
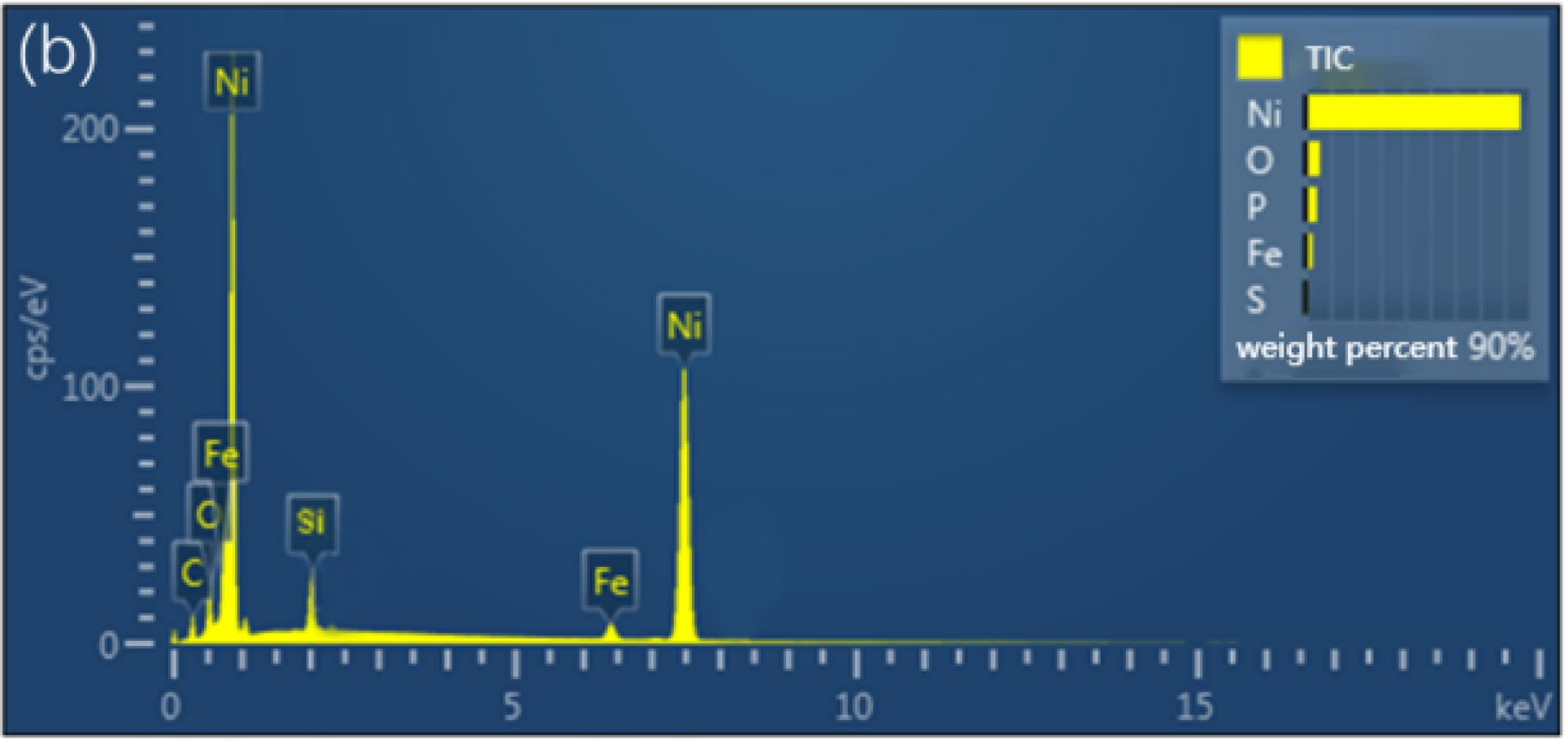
EDS spectra after room-temperature adsorption on silanized surface.

Moreover, electron energy spectrum analysis was conducted to scrutinize elemental shifts on the surface subsequent to adsorption (Fig. 6). This analysis indicated the absence of sulfur elements, affirming the absence of chemical adsorption of sulfur-containing elements on the silanized surface. These findings align with the theoretical study’s conclusions, suggesting the non-adsorption of the four sulfide gas molecules on the surface.

**Fig. 7:**
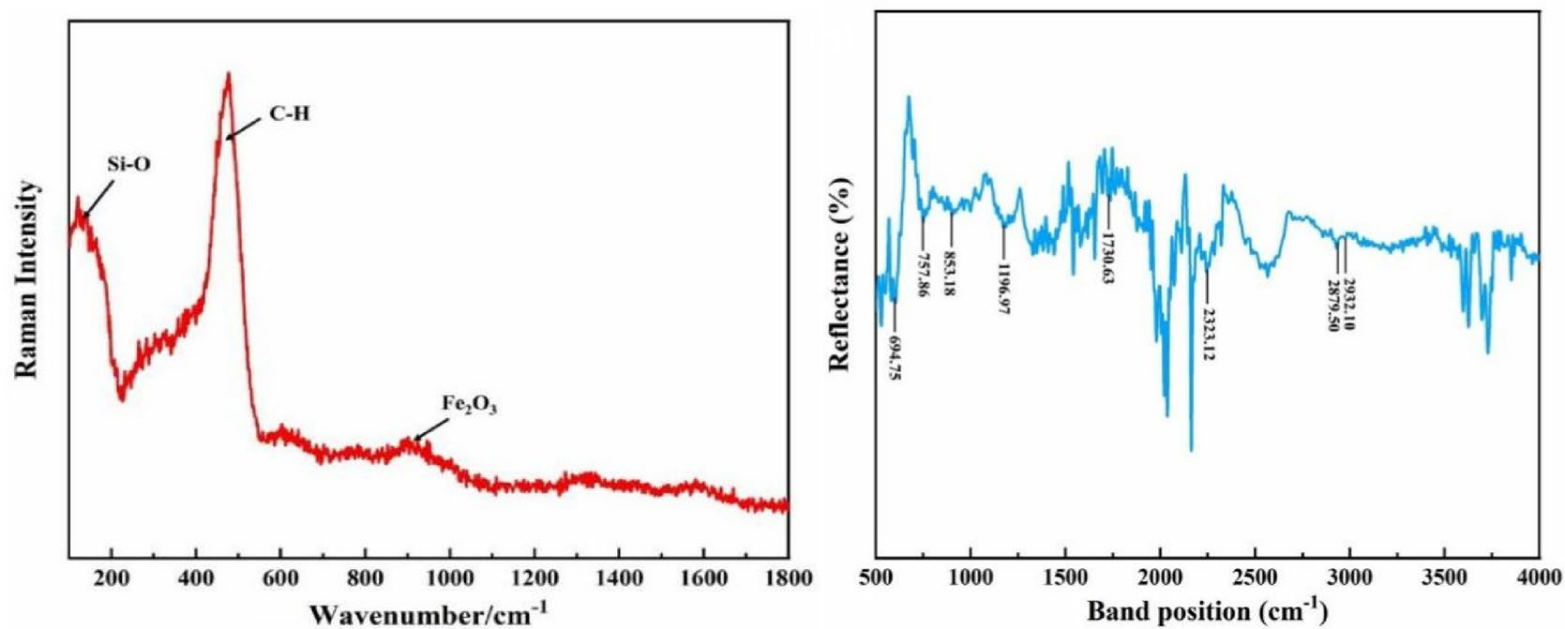
(**a**) Raman spectra of silanized stainless steel after adsorption. (**b**) Infrared spectra of silanized stainless steel surface after adsorption.

Raman spectroscopy (Fig. 7a was employed to explore the alterations in functional groups on the surface following adsorption. Peaks observed at 175 cm^−1^ and 476 cm^−1^ aligned with typical C-H and Si-O bonds, predominant components of silanized molecules. Notably, no vibrational peaks linked to Fe-S bonds or other sulfur atom-related bonds were identified. This absence indicates the absence of chemical bonding between the silanized surface and sulfide molecules. These findings corroborate the theoretical analysis, affirming the effective inhibition of sulfide adsorption by the silanized surface.

The theoretical analysis of the adsorption mechanism revealed that in the calculations for the adsorption of the four sulfide molecules on the silanized surface, the configuration positioned each sulfide gas molecule at a distance from the surface atoms. The surface’s functional groups showcased a repulsive effect on the gas molecules. Moreover, there was no observed transfer of charge between the gas molecules and the surface atoms. Consequently, the spectroscopic experiments provided further confirmation that no fresh sulfur-containing chemical bonds were established on the surface.

For a comprehensive investigation into potential surface chemical reactions post-adsorption, an infrared spectroscopy test was carried out on the silanized stainless steel surface (Fig. 7b. The results unveiled several absorption peaks and their corresponding vibrations:

- Peaks at 2932.10 cm^−1^ and 2879.50 cm^−1^ align with the asymmetric and symmetric stretching vibrations of methylene (-CH_2_-) and methyl (-CH_3_) groups in silanes, originating from methoxy and ethoxy groups within the silanes.
- The 2323.12 cm^−1^ peak is attributed to the asymmetric stretching vibration of CO_2_ in the environment.
- An absorption peak at 1730.63 cm^−1^ represents the stretching vibration of the carbonyl double bond (C=O).
- The peak at 1196.97 cm^−1^ is a characteristic of Si-O-Si antisymmetric stretching and bending vibrations, originating from silane and silica sol. This suggests condensation reactions between monomers post-hydrolysis of silane, with silica sol contributing to film formation.
- At 853.18 cm^−1^, a peak indicates the stretching vibration of Si-O-Fe, confirming stable chemical bond formation between silane monomers and hydroxyl groups on the metal interface. This results in a tightly covered silane film layer on the substrate surface.
- Peaks at 757.86 cm^−1^ and 694.75 cm^−1^ represent Si-C bond stretching vibrations.

Notably, no characteristic peaks associated with sulfides or their decomposition products were discernible in the spectrum. This absence validates the conclusion that sulfides did not adsorb onto the silanized stainless steel surface.

The infrared spectroscopy analysis reveals that silane monomers establish Si-O-Fe chemical bonds with metal hydroxyl groups on the substrate interface through dehydration condensation. Additionally, these monomers undergo dehydration condensation amongst themselves, generating Si-O-Si bonds that culminate in a networked cross-linked structure. This process reinforces the bonding between silane and the metal interface, elevating the film layer’s density and significantly bolstering its protective and anti-adsorption properties. Functioning as an inert layer on the stainless steel surface, the silanized coating exhibits a repulsive effect toward polar gas molecules, thereby achieving exceptional anti-adsorption performance.

### Macroscopic adsorption verification experiment

**Fig. 8:**
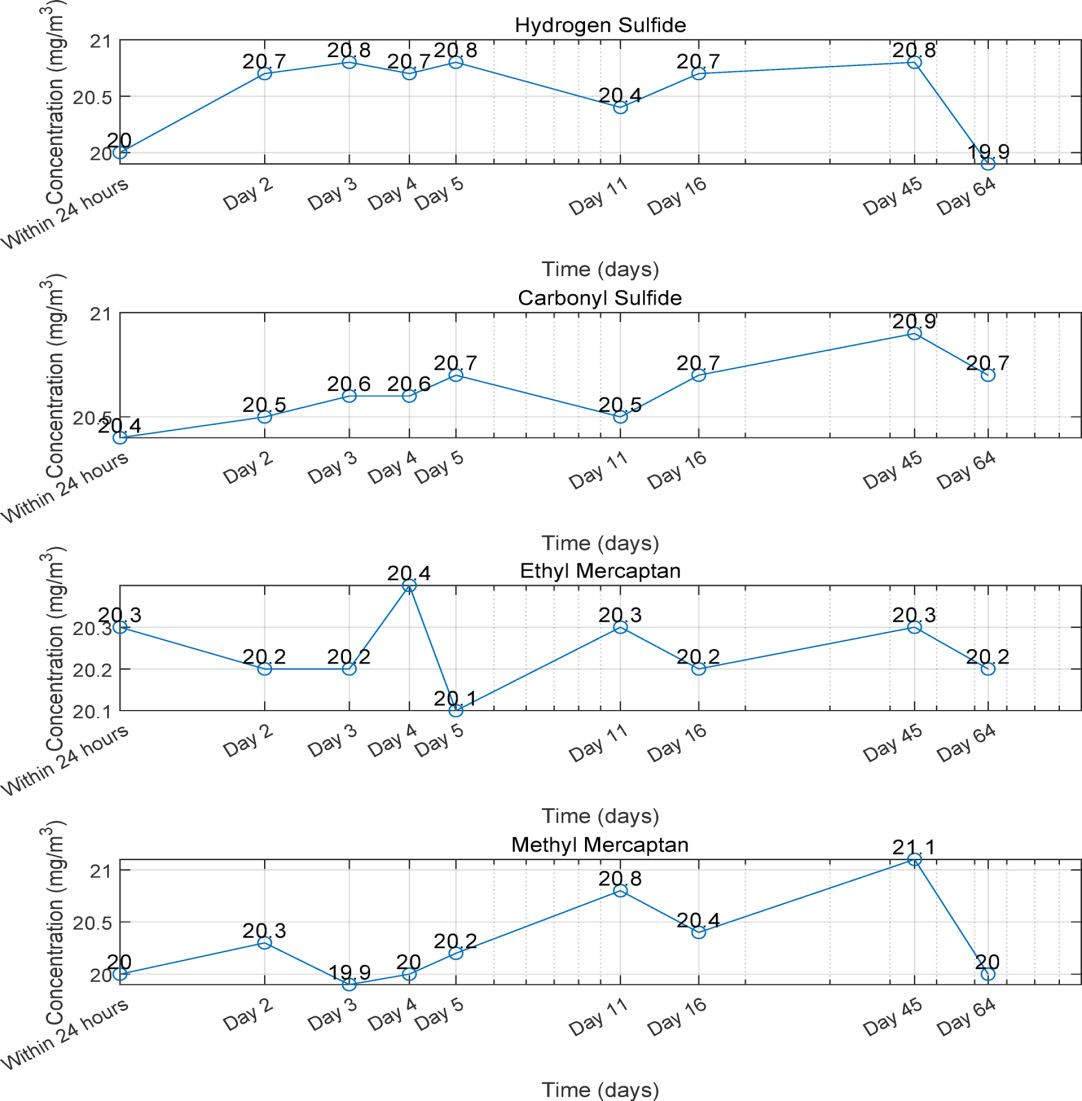
Trend of sulfide content over time (inert steel bottles filled with 20 mg-level sulfide standard gas).

To validate the prognostications of the adsorption mechanism, an experiment was conducted to gauge the adsorption of four sulfides within a silanized stainless steel bottle. A 1-liter silanized stainless steel bottle was filled with a nitrogen standard substance containing hydrogen sulfide, carbonyl sulfide, methanethiol, and ethanethiol (hydrogen sulfide 20.5 mg/m^3^, carbonyl sulfide 20.6 mg/m^3^, methanethiol 20.2 mg/m^3^, ethanethiol 20.3 mg/m^3^). The sulfur content of the four compounds was then measured at nine different time points (within 24 h, and on the 2nd, 3rd, 4th, 5th, 11th, 16th, 45th, and 64th days) using the TCD gas chromatography method. Each data set underwent five measurements, and the average values were calculated. The average measured values for each sulfide were within the ranges of hydrogen sulfide (20.0-20.8) mg/m^3^, carbonyl sulfide (20.4–20.9) mg/m^3^, ethanethiol (20.1–20.4) mg/m^3^, and methanethiol (19.9–21.1) mg/m^3^. The results are depicted in Fig. 8.

After a 60-day adsorption test, the experimental findings reveal relatively stable concentrations of the four sulfide gases, exhibiting only slight fluctuations. Analysis of the adsorption mechanism denotes that these sulfide molecules lack the capacity for spontaneous adsorption on the silanized surface, remaining distanced from the adsorption sites in the configuration calculations. This reaffirms the substantial anti-adsorption characteristics of the silanized surface against polar sulfide molecules, aligning seamlessly with the theoretical analysis.

## Conclusion

The research pioneers the utilization of Density Functional Theory (DFT) to explore the inhibitory effects of silanized stainless steel on sulfide adsorption within natural gas. The main conclusions drawn from this this work are as follows:

Through DFT calculations, it was revealed for the first time that the adsorption energies of hydrogen sulfide, thionyl chloride, methanethiol, and ethanethiol on silanized stainless steel surfaces are 1.55 eV, 1.87 eV, 1.56 eV, and 1.60 eV, respectively. All these values are positive, indicating the need for external energy input during adsorption, making spontaneous adsorption improbable. Mulliken charge population analysis reinforces this by indicating minimal charge transfer between the four sulfur compounds and the silanized stainless steel surface, suggesting a lack of substantial interaction and ruling out dissociative chemical adsorption.

Microscopic experimental findings corroborate this, demonstrating no observed adsorption of sulfides on the silanized stainless steel surface. The silanization process establishes strong Si-O-Fe bonds, ensuring robust adhesion between the coating and the stainless steel substrate. Concurrently, the formation of Si-O-Si bonds within the coating engenders a stable cross-linked network structure. This silanized coating acts as an inert protective layer, significantly bolstering the surface’s resilience against external environments.

Macroscopic experimental validation over a continuous 60-day adsorption test affirms the stability of the concentrations of the four sulfide gases with minor fluctuations. This underscores the substantial anti-adsorption characteristics of surfaces treated with silanization against polar sulfide molecules. These empirical findings align closely with theoretical predictions, further affirming the efficacy of silanization in impeding sulfide adsorption.

## Data Availability

The datasets used and/or analysed during the current study available from the corresponding author on reasonable request.
